# Supplementary material for: A yeast phenomic model for the influence of Warburg metabolism on genetic buffering of doxorubicin
Source: Cancer Metab. 2019 Oct 23;7:9. doi: 10.1186/s40170-019-0201-3 (PMC6806529; doi:10.1186/s40170-019-0201-3)
Supplement: Supplementary file 9 — Additional file 9. GO term-specific heatmaps for REMc/GTF-enriched clusters. GO term-specific heatmaps for significant GO process terms were generated as described in methods and Figs. 3 and 4. Any related child terms are presented in subsequent pages of the parent file name. GO terms with more than 100 children, with 2 or fewer genes annotated to the term, or a file size over 300KB are not shown. All heatmaps are generated with the same layout (see Figs. 3 and 4). [file 40170_2019_201_MOESM9_ESM.bz2 › Additional_File9_GOTermHeatmaps/Additional_File9_GOTermHeatmap/rRNA_metabolic_process.pdf]

rRNA metabolic process

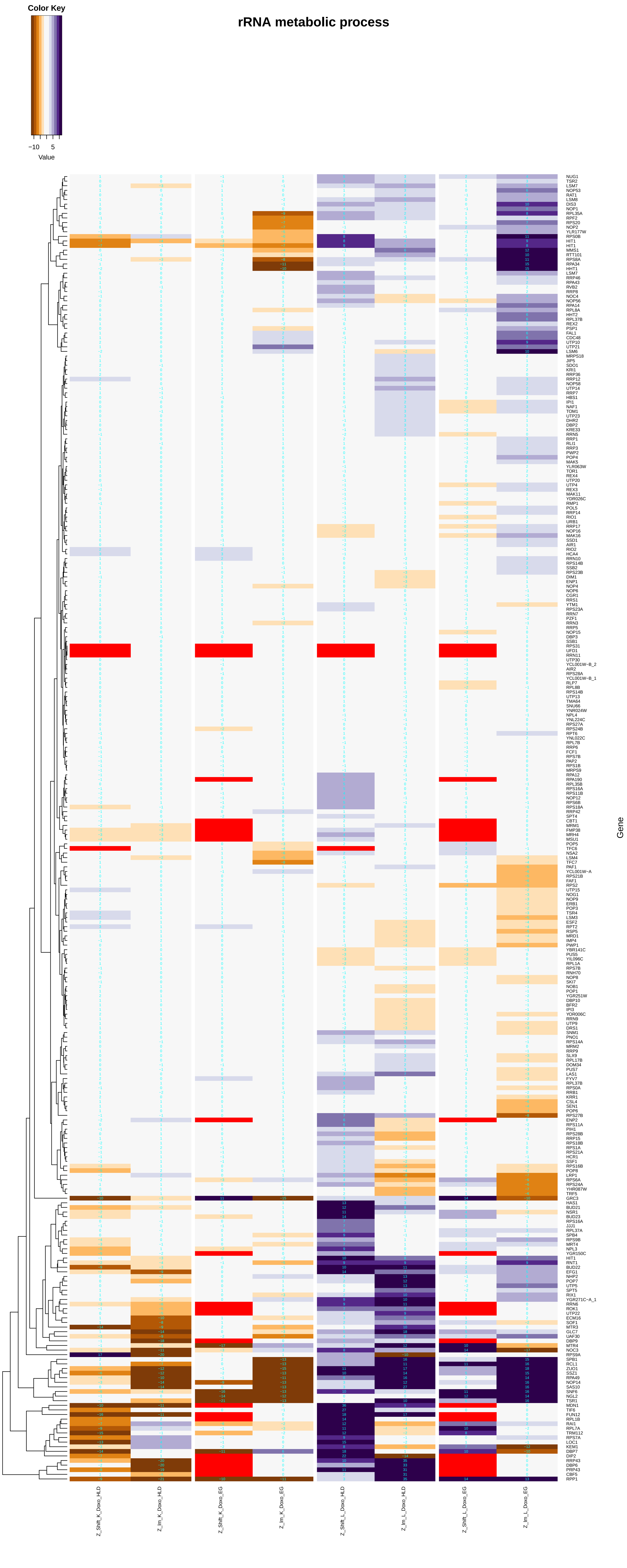

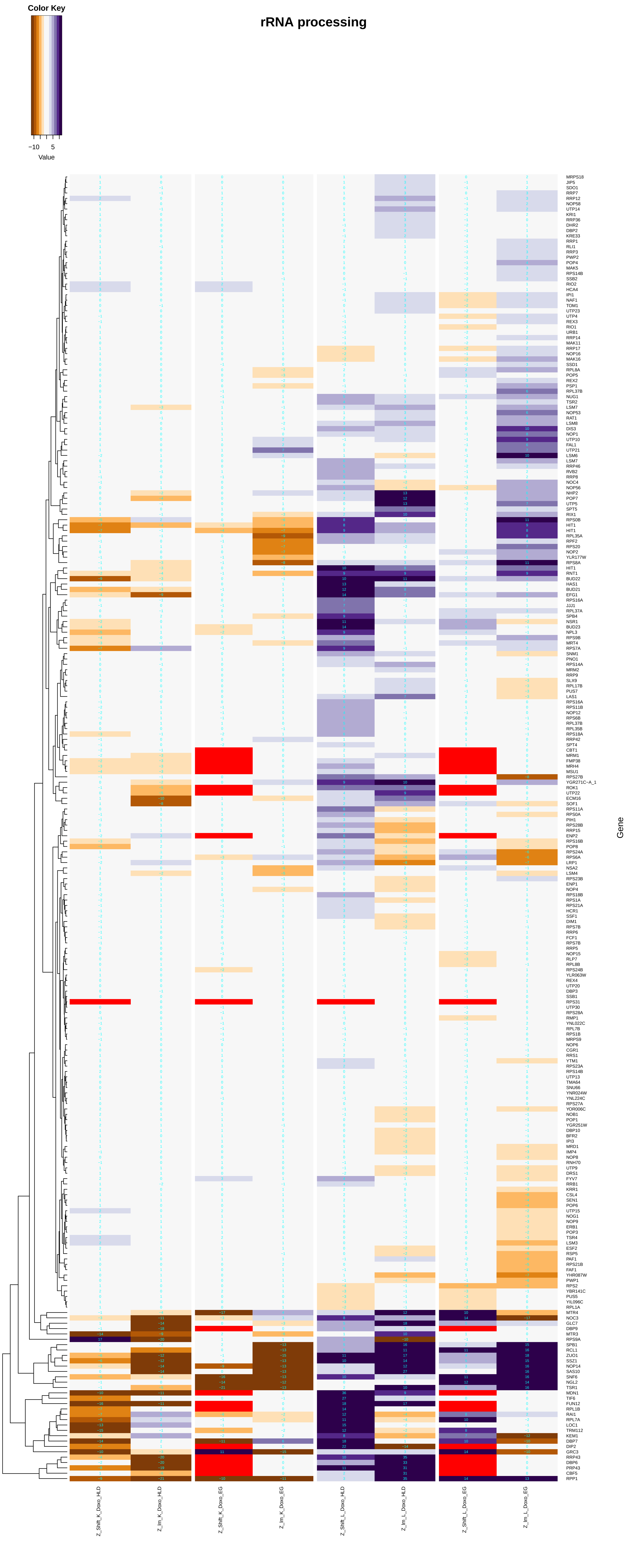
rRNA processing
Color Key
−10
5
Value
Z_Shift_K_Doxo_HLD
Z_Im_K_Doxo_HLD
Z_Shift_K_Doxo_EG
Z_Im_K_Doxo_EG
Z_Shift_L_Doxo_HLD
Z_Im_L_Doxo_HLD
Z_Shift_L_Doxo_EG
Z_Im_L_Doxo_EG
Gene

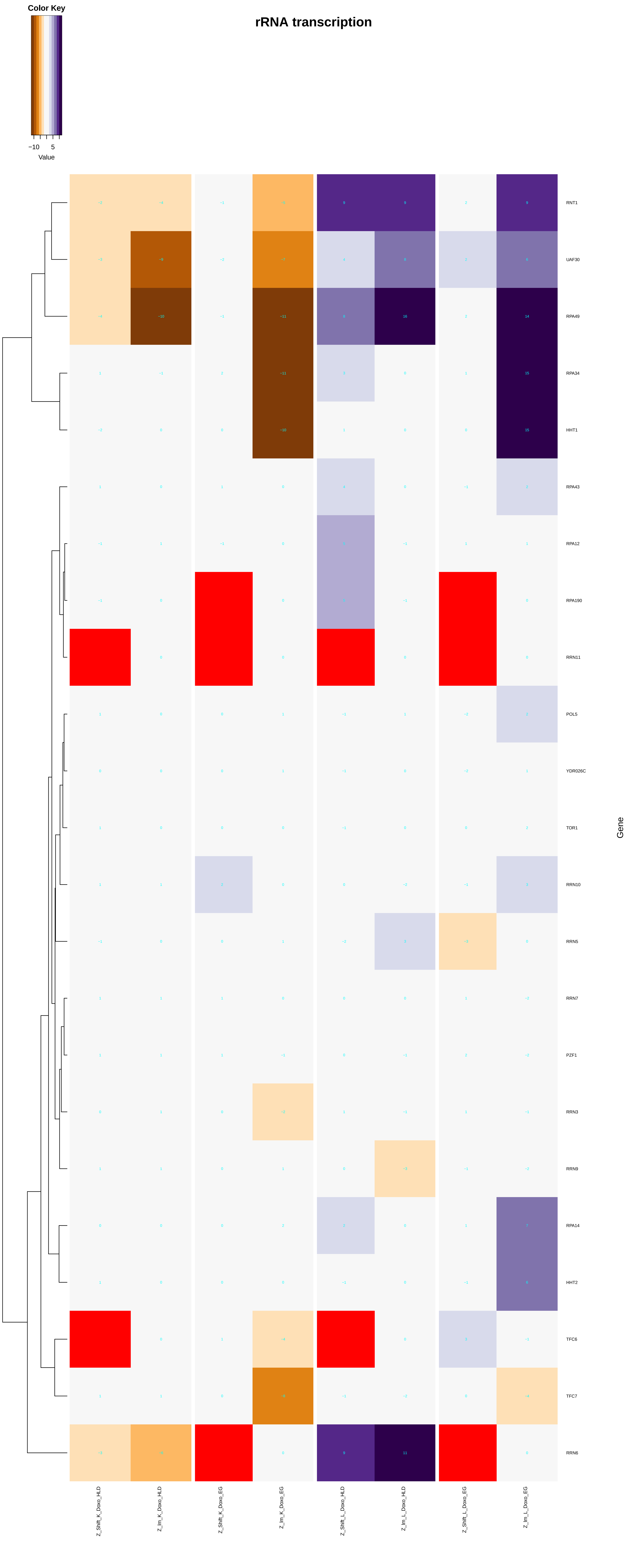

rRNA transcription
Color Key
−10
5
Value
RNT1
UAF30
RPA49
RPA34
HHT1
RPA43
RPA12
RPA190
RRN11
POL5
YDR026C
TOR1
RRN10
RRN5
RRN7
PZF1
RRN3
RRN9
RPA14
HHT2
TFC6
TFC7
RRN6
Gene
Z_Shift_K_Doxo_HLD
Z_lm_K_Doxo_HLD
Z_Shift_K_Doxo_EG
Z_lm_K_Doxo_EG
Z_Shift_L_Doxo_HLD
Z_lm_L_Doxo_HLD
Z_Shift_L_Doxo_EG
Z_lm_L_Doxo_EG

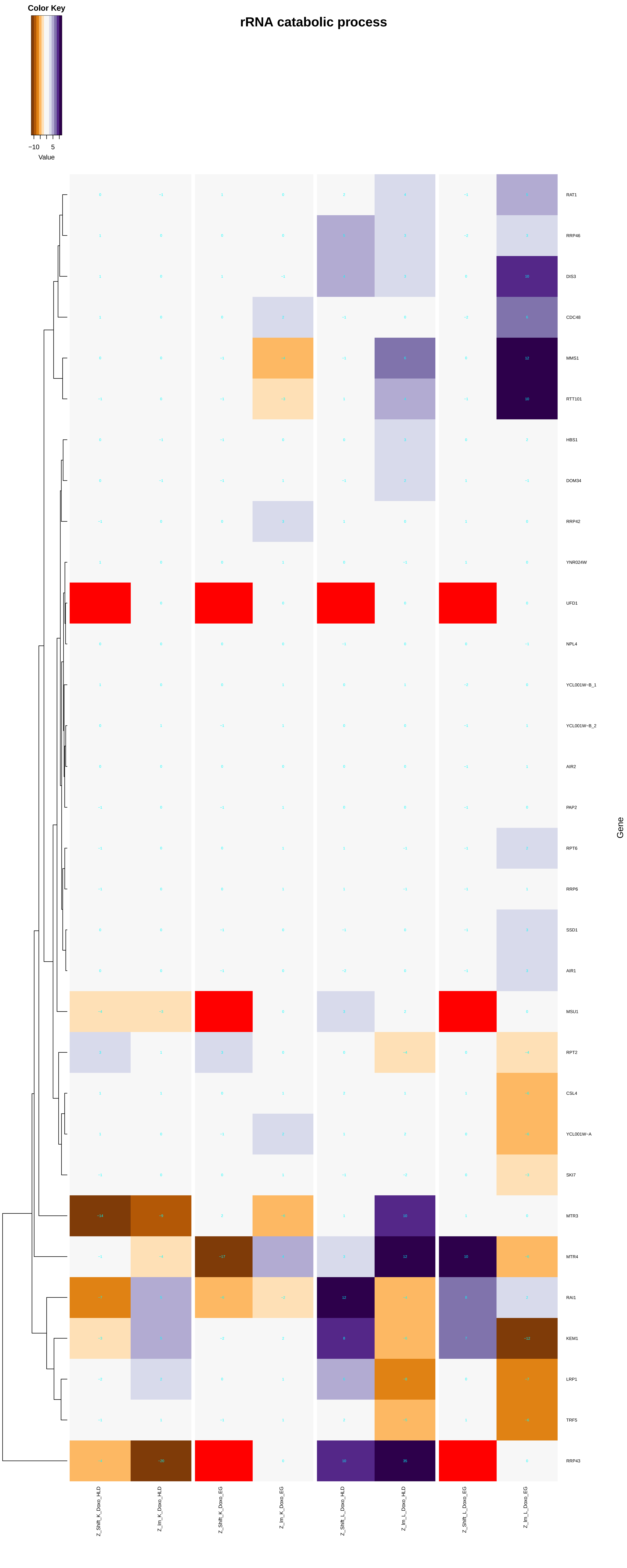

Color Key
−10
5
Value
rRNA catabolic process
RAT1
RRP46
DIS3
CDC48
MMS1
RTT101
HBS1
DOM34
RRP42
YNR024W
UFD1
NPL4
YCL001W−B_1
YCL001W−B_2
AIR2
PAP2
RPT6
RRP6
SSD1
AIR1
MSU1
RPT2
CSL4
YCL001W−A
SKI7
MTR3
MTR4
RAI1
KEM1
LRP1
TRF5
RRP43
Gene
Z_Shift_K_Doxo_HLD
Z_lm_K_Doxo_HLD
Z_Shift_K_Doxo_EG
Z_lm_K_Doxo_EG
Z_Shift_L_Doxo_HLD
Z_lm_L_Doxo_HLD
Z_Shift_L_Doxo_EG
Z_lm_L_Doxo_EG

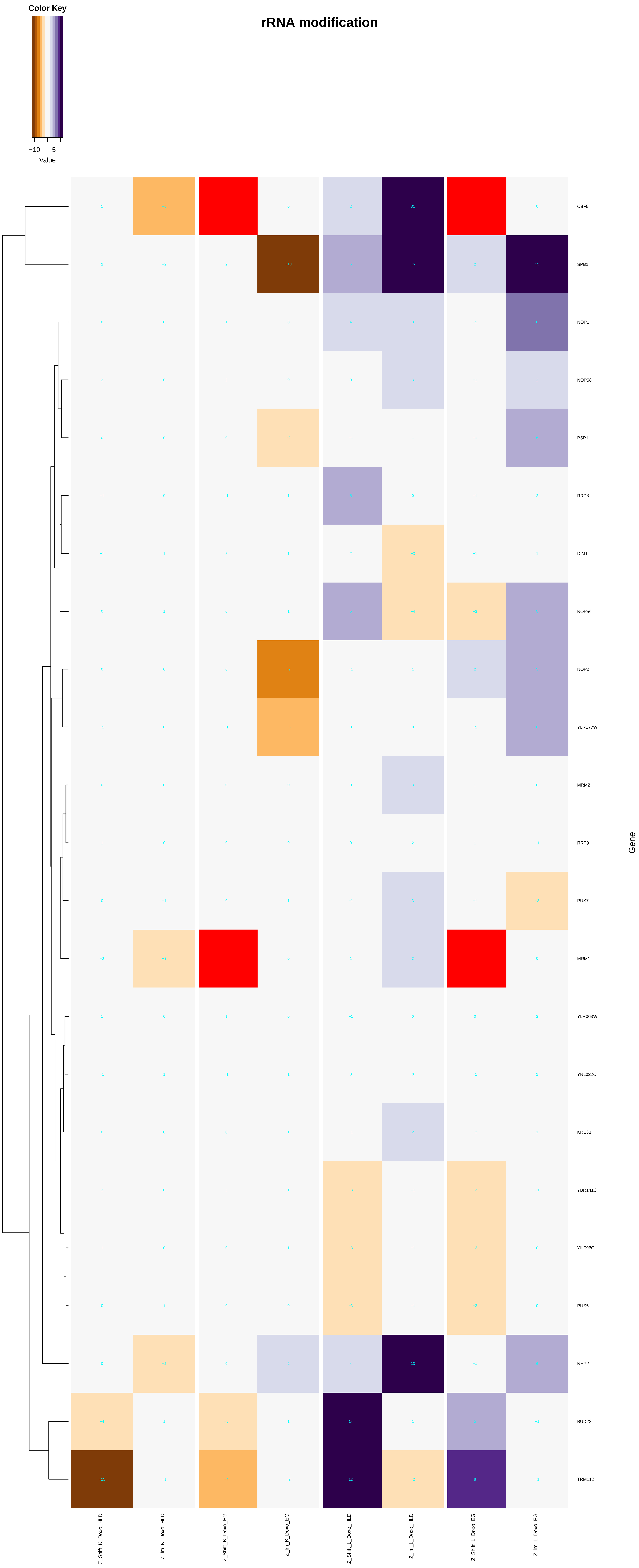

rRNA modification
Color Key
−10
5
Value
CBF5
SPB1
NOP1
NOP58
PSP1
RRP8
DIM1
NOP56
NOP2
YLR177W
MRM2
RRP9
PUS7
MRM1
YLR063W
YNL022C
KRE33
YBR141C
YIL096C
PUS5
NHP2
BUD23
TRM112
Gene
Z_Shift_K_Doxo_HLD
Z_Im_K_Doxo_HLD
Z_Shift_K_Doxo_EG
Z_Im_K_Doxo_EG
Z_Shift_L_Doxo_HLD
Z_Im_L_Doxo_HLD
Z_Shift_L_Doxo_EG
Z_Im_L_Doxo_EG

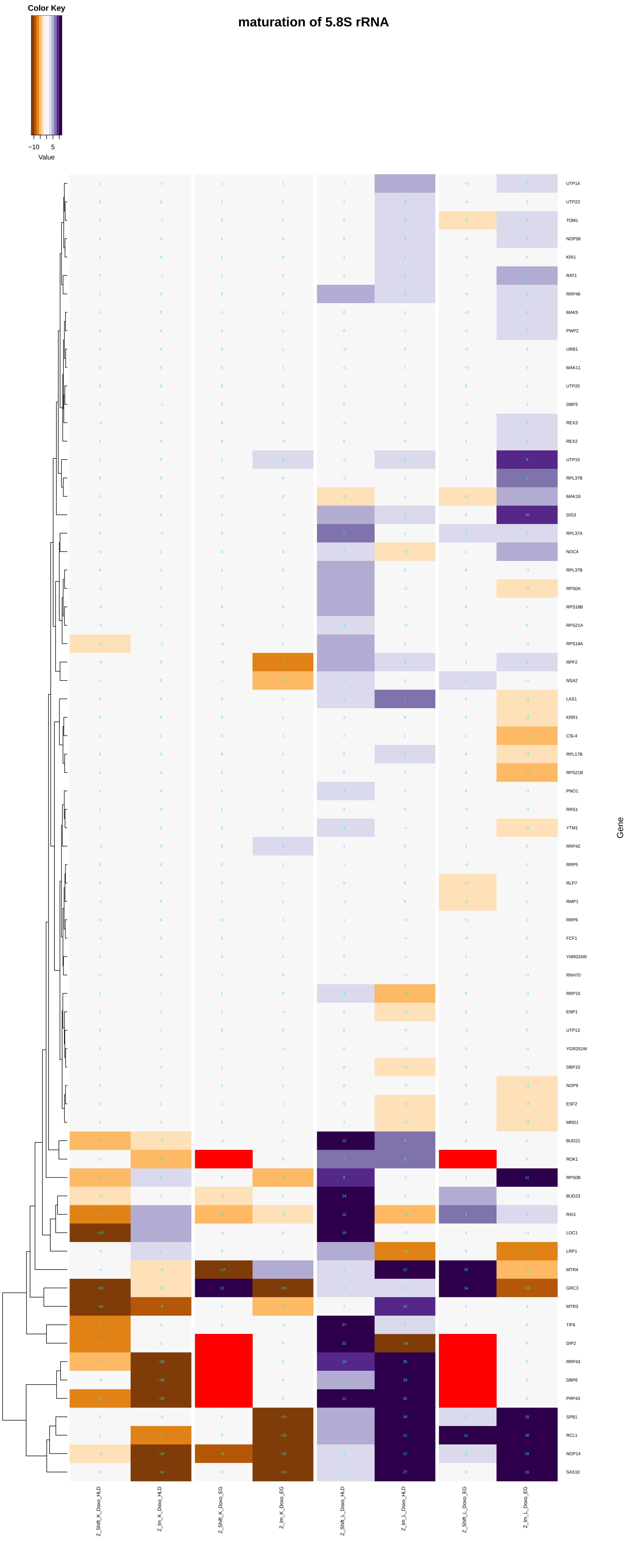
Color Key
−10 5
Value
maturation of 5.8S rRNA
UTP14
UTP23
TOM1
NOP58
KRI1
RAT1
RRP46
MAK5
PWP2
URB1
MAK11
UTP20
DBP3
REX3
REX2
UTP10
RPL37B
MAK16
DIS3
RPL37A
NOC4
RPL37B
RPS0A
RPS18B
RPS21A
RPS18A
RPF2
NSA2
LAS1
KRR1
CSL4
RPL17B
RPS21B
PNO1
RRS1
YTM1
RRP42
RRP5
RLP7
RMP1
RRP6
FCF1
YNR024W
RNH70
RRP15
ENP1
UTP13
YGR251W
DBP10
NOP9
ESF2
MRD1
BUD21
ROK1
RPS0B
BUD23
RAI1
LOC1
LRP1
MTR4
GRC3
MTR3
TIF6
DIP2
RRP43
DBP6
PRP43
SPB1
RCL1
NOP14
SAS10
Gene
Z_Shift_K_Doxo_HLD
Z_Im_K_Doxo_HLD
Z_Shift_K_Doxo_EG
Z_Im_K_Doxo_EG
Z_Shift_L_Doxo_HLD
Z_Im_L_Doxo_HLD
Z_Shift_L_Doxo_EG
Z_Im_L_Doxo_EG

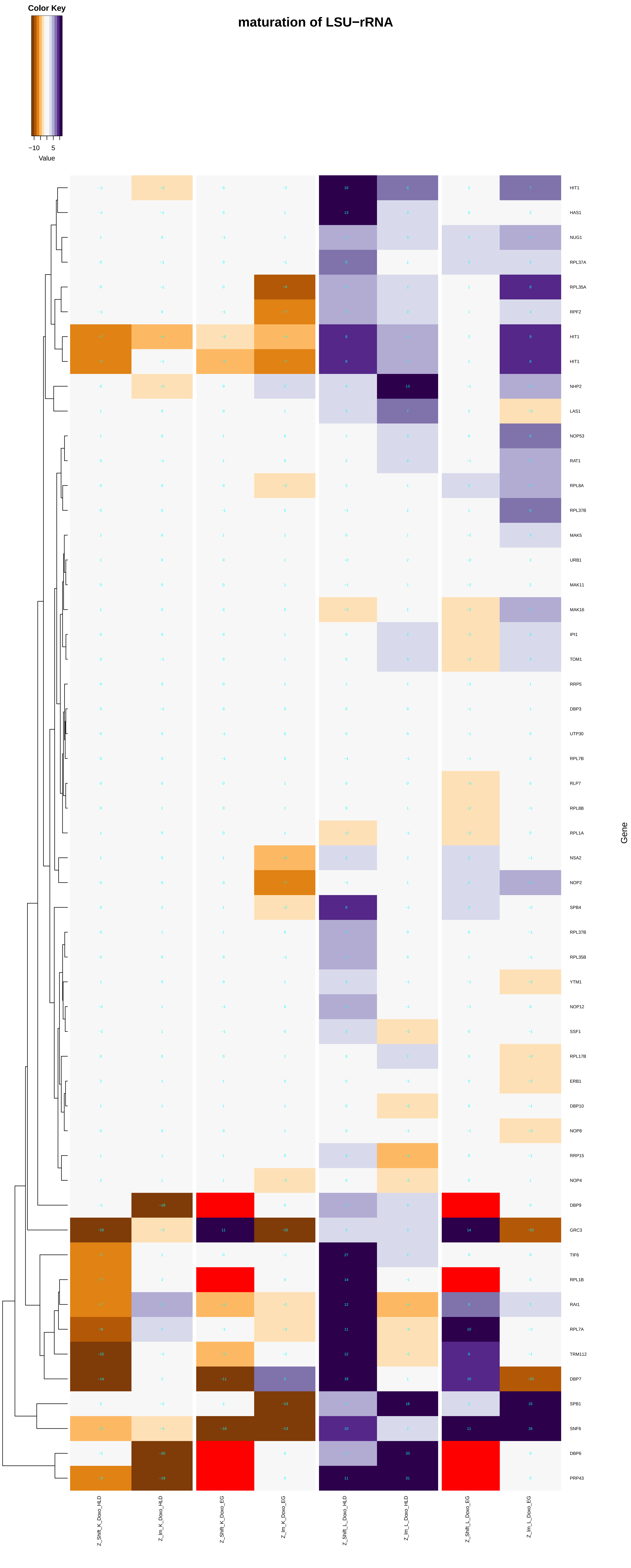

maturation of LSU−rRNA
Color Key
−10
5
Value
HIT1
HAS1
NUG1
RPL37A
RPL35A
RPF2
HIT1
HIT1
NHP2
LAS1
NOP53
RAT1
RPL8A
RPL37B
MAK5
URB1
MAK11
MAK16
IPI1
TOM1
RRP5
DBP3
UTP30
RPL7B
RLP7
RPL8B
RPL1A
NSA2
NOP2
SPB4
RPL37B
RPL35B
YTM1
NOP12
SSF1
RPL17B
ERB1
DBP10
NOP8
RRP15
NOP4
DBP9
GRC3
TIF6
RPL1B
RAI1
RPL7A
TRM112
DBP7
SPB1
SNF6
DBP6
PRP43
Gene
Z_Shift_K_Doxo_HLD
Z_Im_K_Doxo_HLD
Z_Shift_K_Doxo_EG
Z_Im_K_Doxo_EG
Z_Shift_L_Doxo_HLD
Z_Im_L_Doxo_HLD
Z_Shift_L_Doxo_EG
Z_Im_L_Doxo_EG

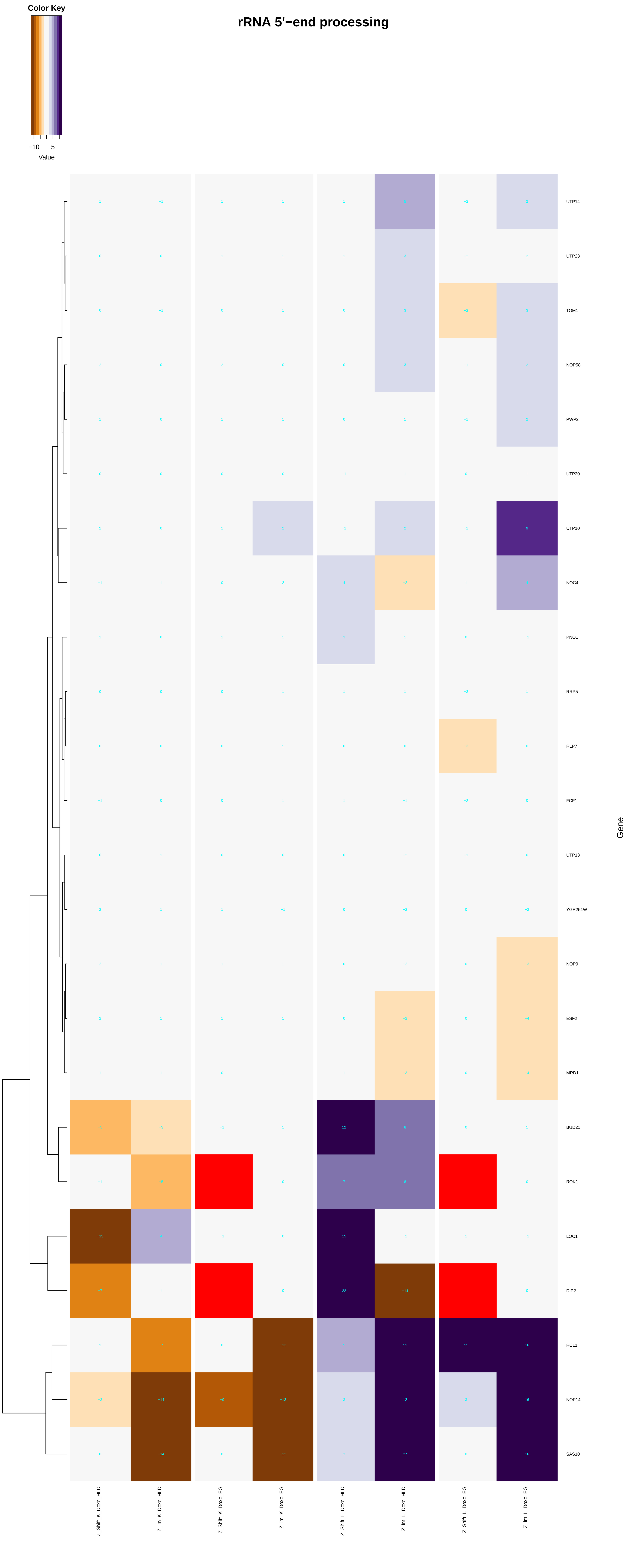
rRNA 5'−end processing
Color Key
−10
5
Value
Gene
UTP14
UTP23
TOM1
NOP58
PWP2
UTP20
UTP10
NOC4
PNO1
RRP5
RLP7
FCF1
UTP13
YGR251W
NOP9
ESF2
MRD1
BUD21
ROK1
LOC1
DIP2
RCL1
NOP14
SAS10
Z_Shift_K_Doxo_HLD
Z_lm_K_Doxo_HLD
Z_Shift_K_Doxo_EG
Z_lm_K_Doxo_EG
Z_Shift_L_Doxo_HLD
Z_lm_L_Doxo_HLD
Z_Shift_L_Doxo_EG
Z_lm_L_Doxo_EG

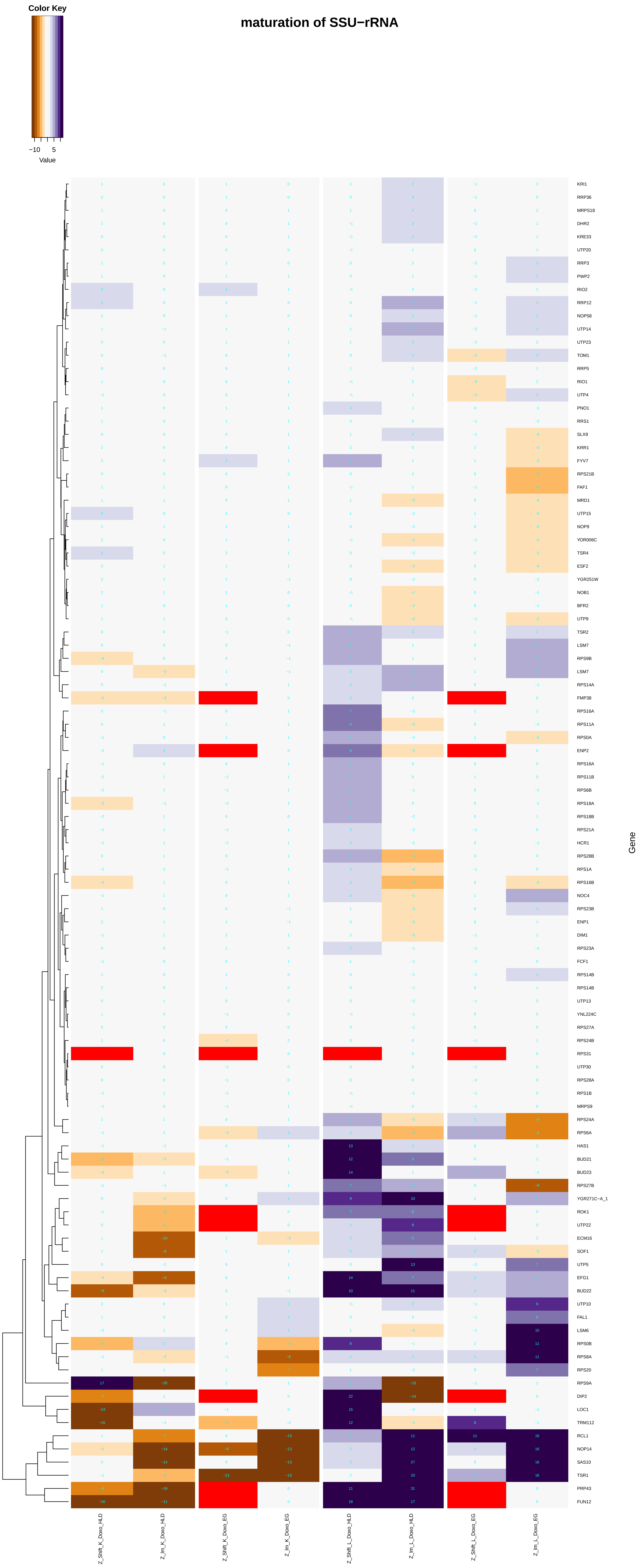

maturation of SSU−rRNA
Color Key
−10
5
Value
KRI1
RRP36
MRPS18
DHR2
KRE33
UTP20
RRP3
PWP2
RIO2
RRP12
NOP58
UTP14
UTP23
TOM1
RRP5
RIO1
UTP4
PNO1
RRS1
SLX9
KRR1
FYV7
RPS21B
FAF1
MRD1
UTP15
NOP9
YOR006C
TSR4
ESF2
YGR251W
NOB1
BFR2
UTP9
TSR2
LSM7
RPS9B
LSM7
RPS14A
FMP38
RPS16A
RPS11A
RPS0A
ENP2
RPS16A
RPS11B
RPS6B
RPS18A
RPS18B
RPS21A
HCR1
RPS28B
RPS1A
RPS16B
NOC4
RPS23B
ENP1
DIM1
RPS23A
FCF1
RPS14B
RPS14B
UTP13
YNL224C
RPS27A
RPS24B
RPS31
UTP30
RPS28A
RPS1B
MRPS9
RPS24A
RPS6A
HAS1
BUD21
BUD23
RPS27B
YGR271C−A_1
ROK1
UTP22
ECM16
SOF1
UTP5
EFG1
BUD22
UTP10
FAL1
LSM6
RPS0B
RPS8A
RPS20
RPS9A
DIP2
LOC1
TRM112
RCL1
NOP14
SAS10
TSR1
PRP43
FUN12
Gene
Z_Shift_K_Doxo_HLD
Z_Im_K_Doxo_HLD
Z_Shift_K_Doxo_EG
Z_Im_K_Doxo_EG
Z_Shift_L_Doxo_HLD
Z_Im_L_Doxo_HLD
Z_Shift_L_Doxo_EG
Z_Im_L_Doxo_EG

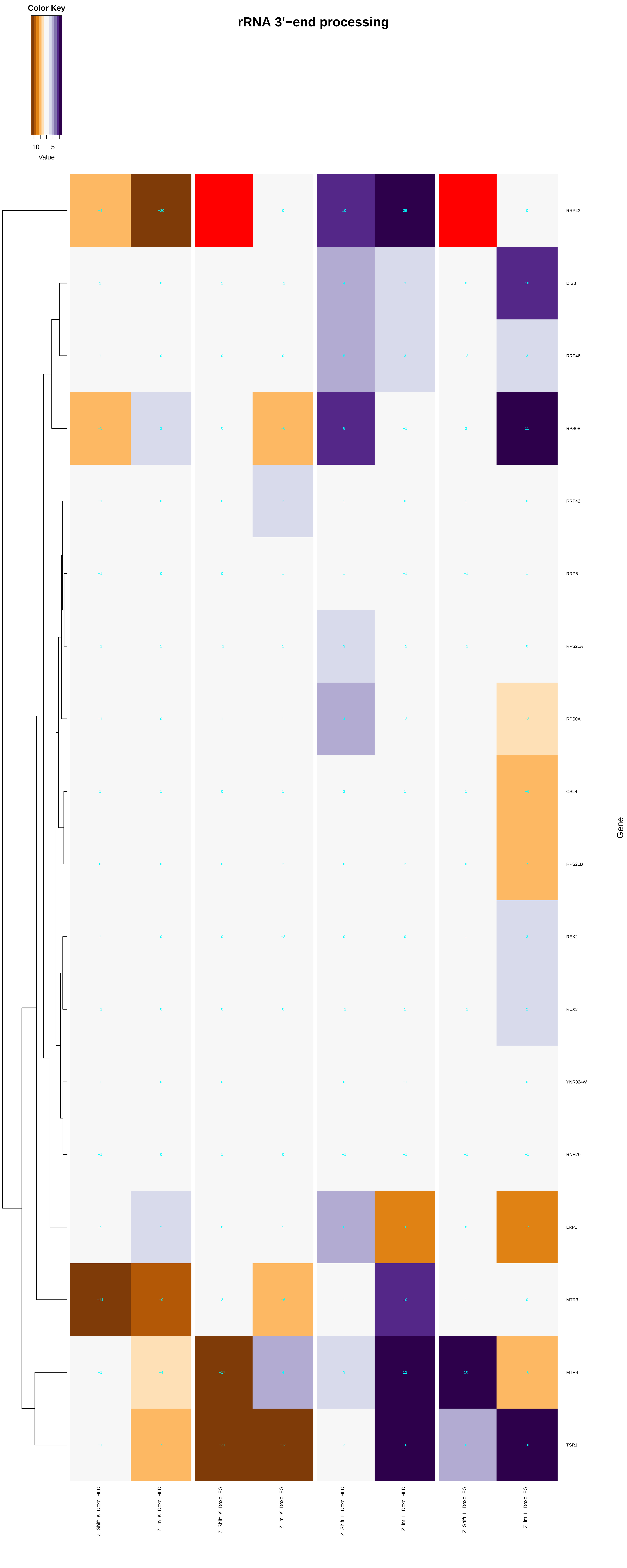

Color Key
−10
5
Value
rRNA 3'−end processing
RRP43
DIS3
RRP46
RPS0B
RRP42
RRP6
RPS21A
RPS0A
CSL4
RPS21B
REX2
REX3
YNR024W
RNH70
LRP1
MTR3
MTR4
TSR1
Gene
Z_Shift_K_Doxo_HLD
Z_Im_K_Doxo_HLD
Z_Shift_K_Doxo_EG
Z_Im_K_Doxo_EG
Z_Shift_L_Doxo_HLD
Z_Im_L_Doxo_HLD
Z_Shift_L_Doxo_EG
Z_Im_L_Doxo_EG

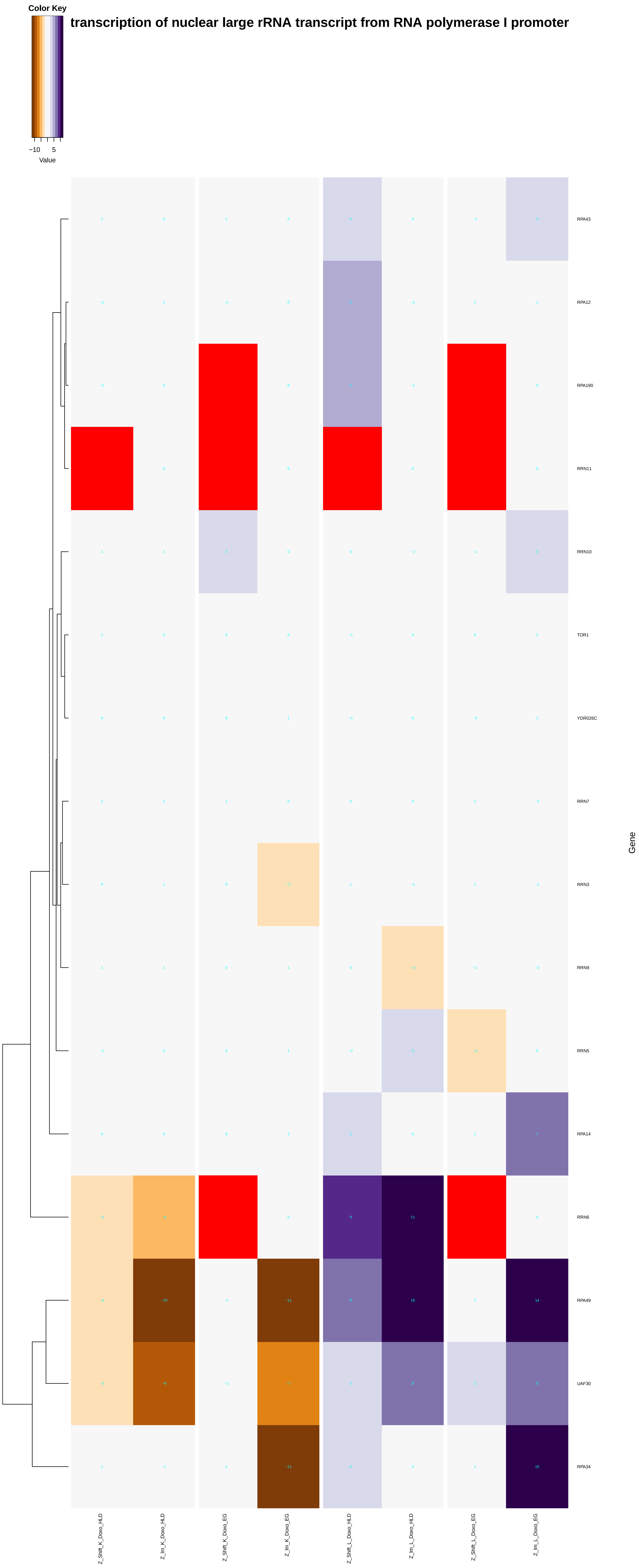

Color Key
−10 5
Value
transcription of nuclear large rRNA transcript from RNA polymerase I promoter
RPA43
RPA12
RPA190
RRN11
RRN10
TOR1
YDR026C
RRN7
RRN3
RRN9
RRN5
RPA14
RRN6
RPA49
UAF30
RPA34
Gene
Z_Shift_K_Doxo_HLD
Z_lm_K_Doxo_HLD
Z_Shift_K_Doxo_EG
Z_lm_K_Doxo_EG
Z_Shift_L_Doxo_HLD
Z_lm_L_Doxo_HLD
Z_Shift_L_Doxo_EG
Z_lm_L_Doxo_EG

5S class rRNA transcription from RNA polymerase III type 1 promoter

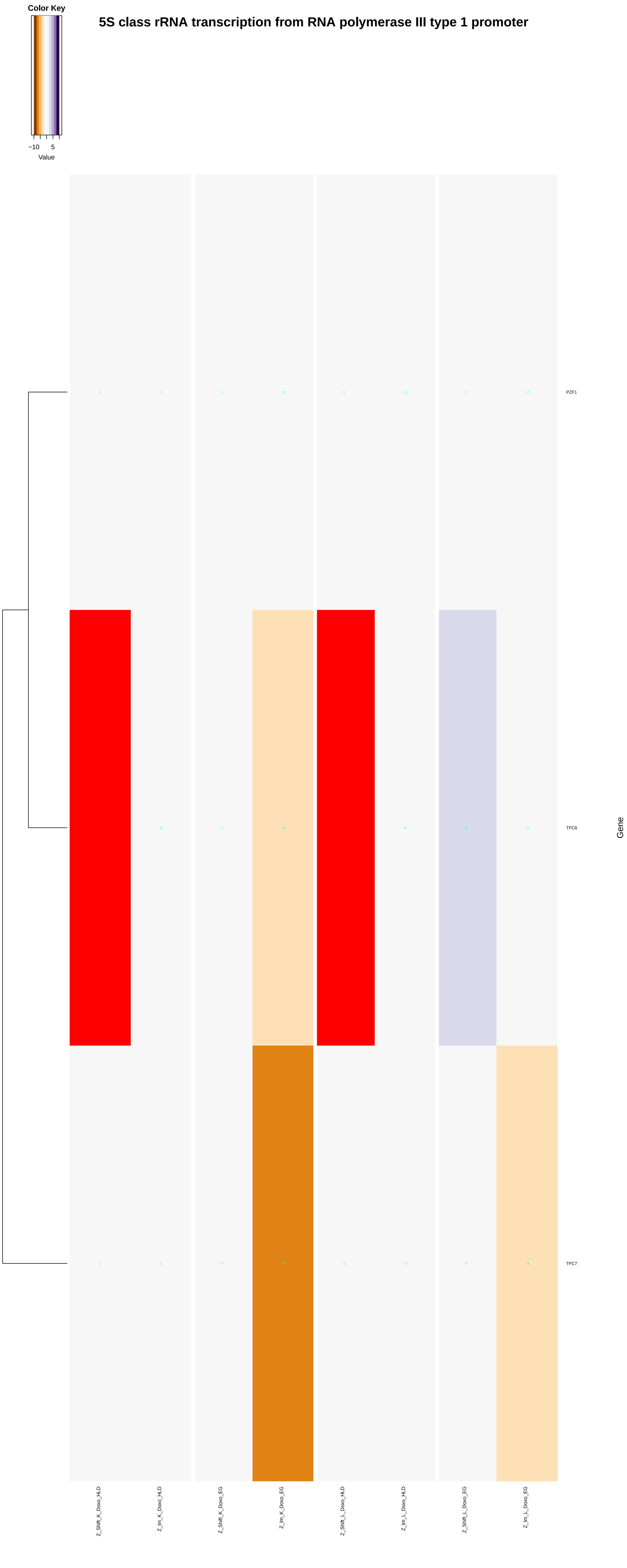

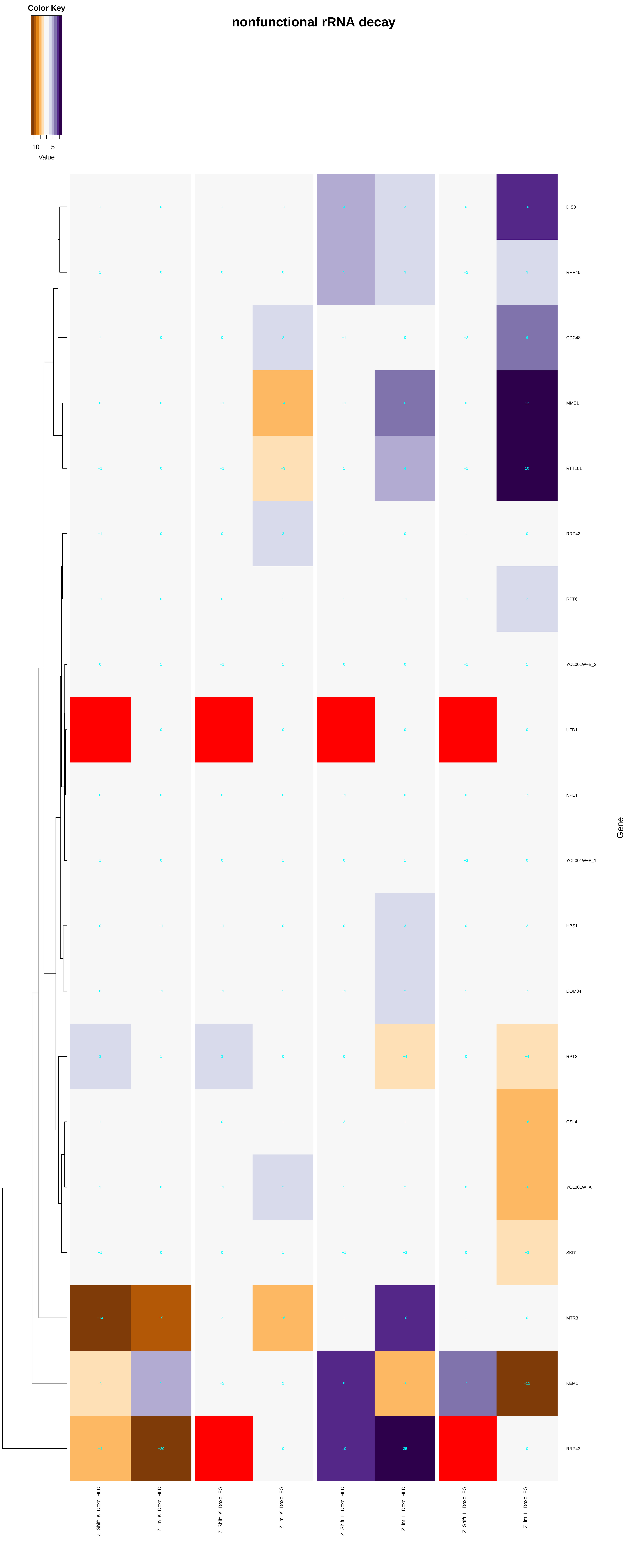

nonfunctional rRNA decay
Color Key
−10
5
Value
Gene
DIS3
RRP46
CDC48
MMS1
RTT101
RRP42
RPT6
YCL001W−B_2
UFD1
NPL4
YCL001W−B_1
HBS1
DOM34
RPT2
CSL4
YCL001W−A
SKI7
MTR3
KEM1
RRP43
Z_Shift_K_Doxo_HLD
Z_lm_K_Doxo_HLD
Z_Shift_K_Doxo_EG
Z_lm_K_Doxo_EG
Z_Shift_L_Doxo_HLD
Z_lm_L_Doxo_HLD
Z_Shift_L_Doxo_EG
Z_lm_L_Doxo_EG

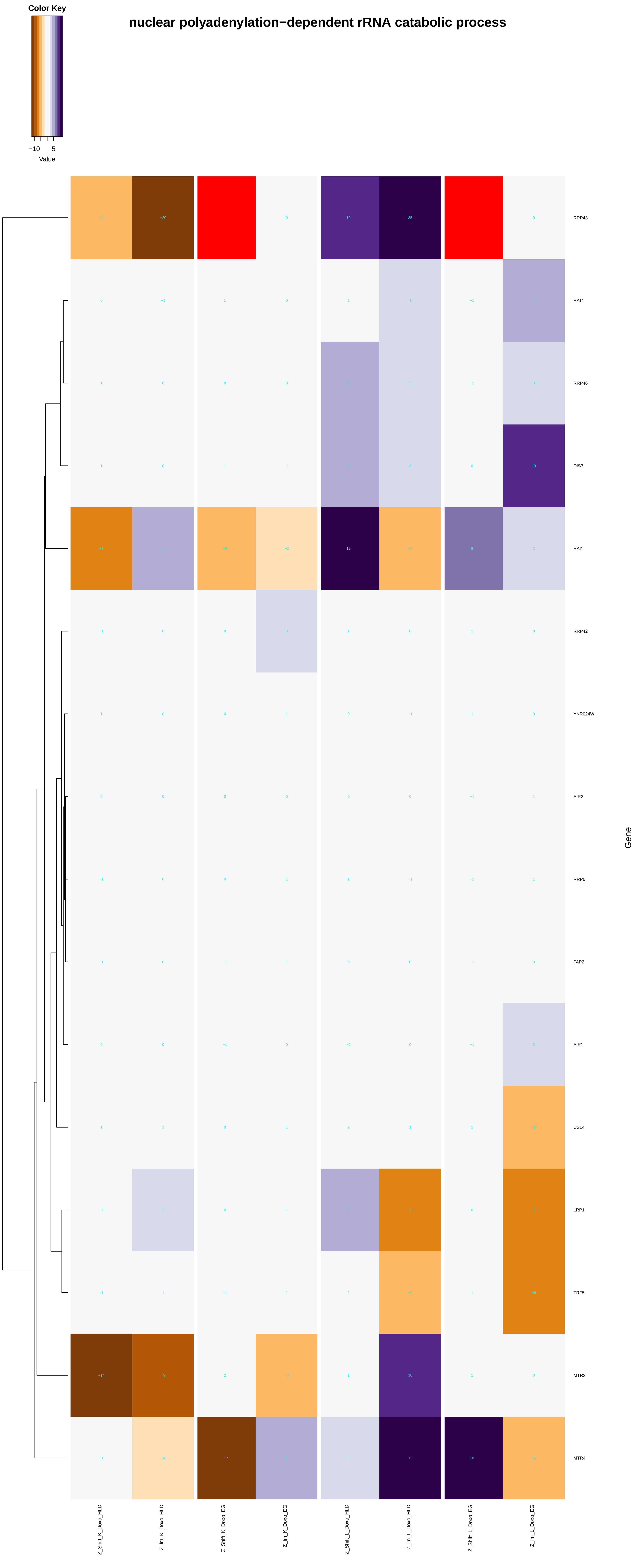

Color Key
−10
5
Value
nuclear polyadenylation−dependent rRNA catabolic process
RRP43
RAT1
RRP46
DIS3
RAI1
RRP42
YNR024W
AIR2
RRP6
PAP2
AIR1
CSL4
LRP1
TRF5
MTR3
MTR4
Gene
Z_Shift_K_Doxo_HLD
Z_Im_K_Doxo_HLD
Z_Shift_K_Doxo_EG
Z_Im_K_Doxo_EG
Z_Shift_L_Doxo_HLD
Z_Im_L_Doxo_HLD
Z_Shift_L_Doxo_EG
Z_Im_L_Doxo_EG

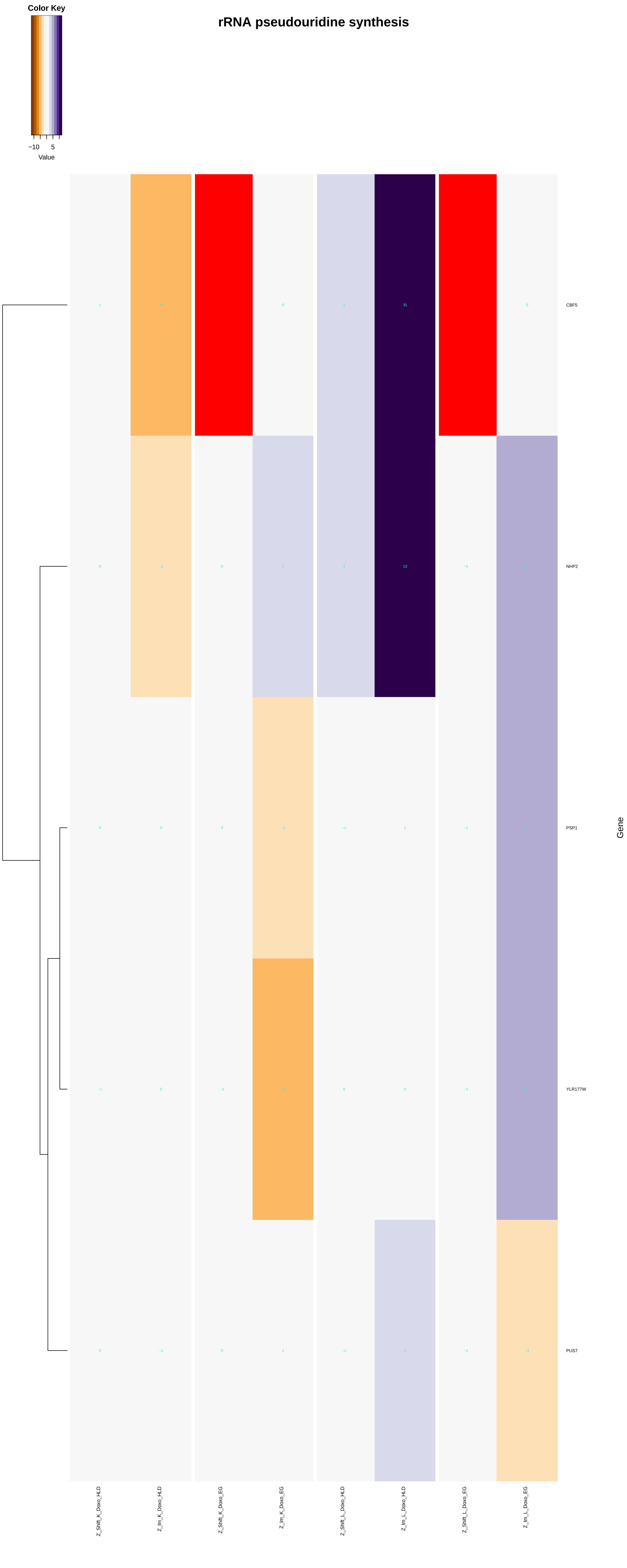

rRNA pseudouridine synthesis
Color Key
−10
5
Value
CBF5
NHP2
PSP1
YLR177W
PUS7
Gene
Z_Shift_K_Doxo_HLD
Z_lm_K_Doxo_HLD
Z_Shift_K_Doxo_EG
Z_lm_K_Doxo_EG
Z_Shift_L_Doxo_HLD
Z_lm_L_Doxo_HLD
Z_Shift_L_Doxo_EG
Z_lm_L_Doxo_EG

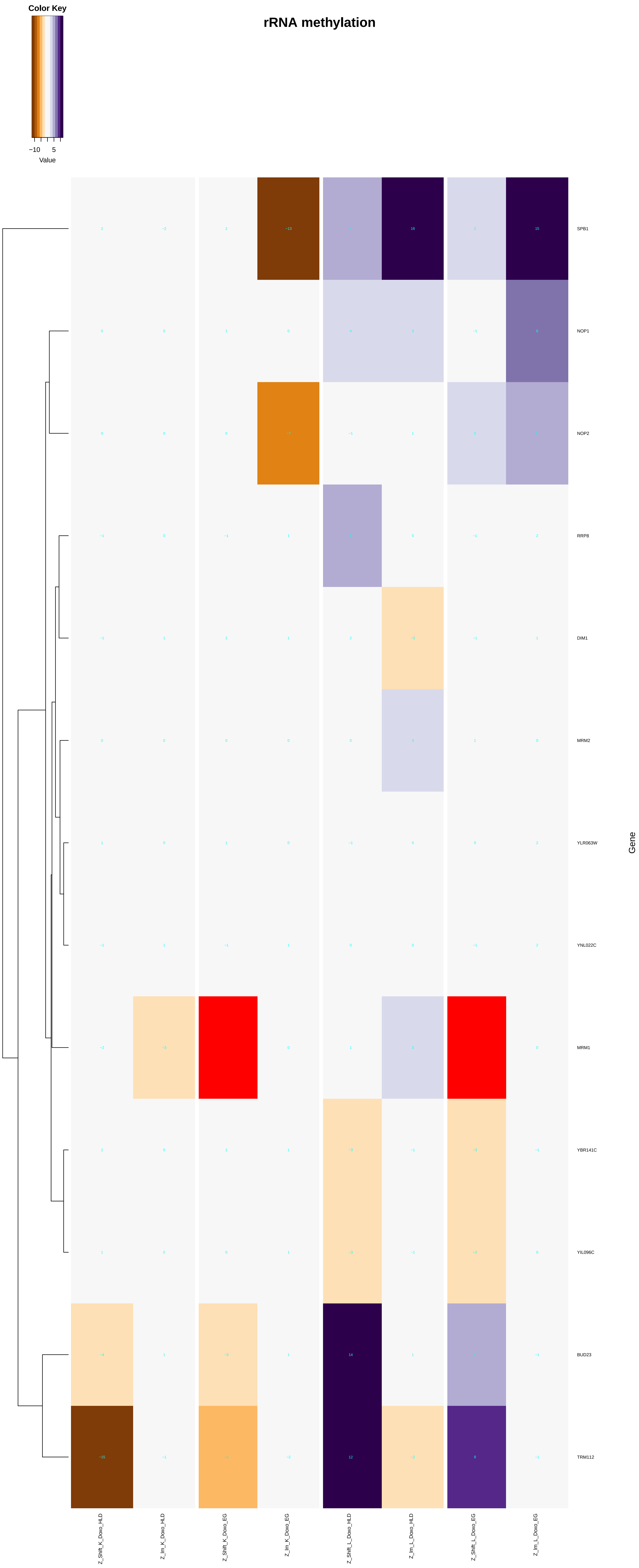

Color Key
−10
5
Value
rRNA methylation
SPB1
NOP1
NOP2
RRP8
DIM1
MRM2
YLR063W
YNL022C
MRM1
YBR141C
YIL096C
BUD23
TRM112
Gene
Z_Shift_K_Doxo_HLD
Z_Im_K_Doxo_HLD
Z_Shift_K_Doxo_EG
Z_Im_K_Doxo_EG
Z_Shift_L_Doxo_HLD
Z_Im_L_Doxo_HLD
Z_Shift_L_Doxo_EG
Z_Im_L_Doxo_EG

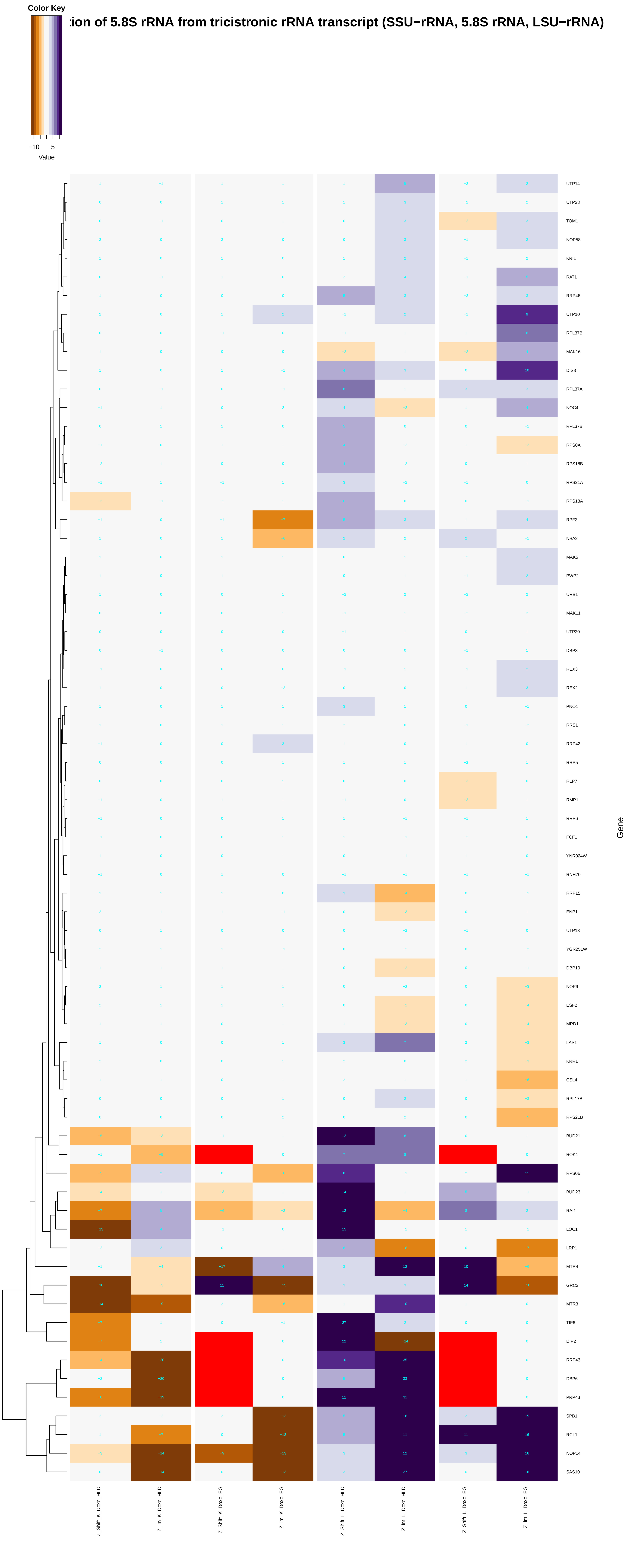
tion of 5.8S rRNA from tricistronic rRNA transcript (SSU−rRNA, 5.8S rRNA, LSU−rRNA)
Color Key
−10 5
Value
UTP14
UTP23
TOM1
NOP58
KRI1
RAT1
RRP46
UTP10
RPL37B
MAK16
DIS3
RPL37A
NOC4
RPL37B
RPS0A
RPS18B
RPS21A
RPS18A
RPF2
NSA2
MAK5
PWP2
URB1
MAK11
UTP20
DBP3
REX3
REX2
PNO1
RRS1
RRP42
RRP5
RLP7
RMP1
RRP6
FCF1
YNR024W
RNH70
RRP15
ENP1
UTP13
YGR251W
DBP10
NOP9
ESF2
MRD1
LAS1
KRR1
CSL4
RPL17B
RPS21B
BUD21
ROK1
RPS0B
BUD23
RAI1
LOC1
LRP1
MTR4
GRC3
MTR3
TIF6
DIP2
RRP43
DBP6
PRP43
SPB1
RCL1
NOP14
SAS10
Gene
Z_Shift_K_Doxo_HLD
Z_Im_K_Doxo_HLD
Z_Shift_K_Doxo_EG
Z_Im_K_Doxo_EG
Z_Shift_L_Doxo_HLD
Z_Im_L_Doxo_HLD
Z_Shift_L_Doxo_EG
Z_Im_L_Doxo_EG

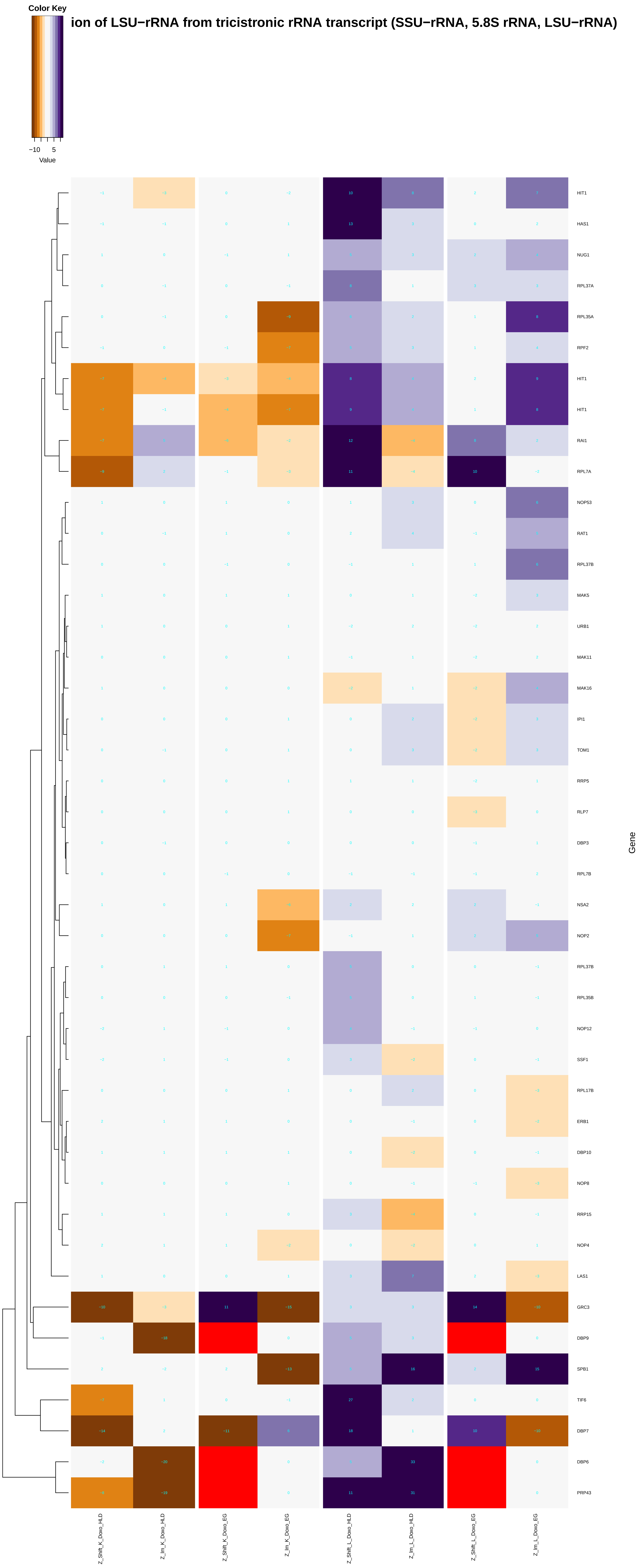
Color Key
−10 5
Value
ion of LSU−rRNA from tricistronic rRNA transcript (SSU−rRNA, 5.8S rRNA, LSU−rRNA)
HIT1
HAS1
NUG1
RPL37A
RPL35A
RPF2
HIT1
HIT1
RAI1
RPL7A
NOP53
RAT1
RPL37B
MAK5
URB1
MAK11
MAK16
IPI1
TOM1
RRP5
RLP7
DBP3
RPL7B
NSA2
NOP2
RPL37B
RPL35B
NOP12
SSF1
RPL17B
ERB1
DBP10
NOP8
RRP15
NOP4
LAS1
GRC3
DBP9
SPB1
TIF6
DBP7
DBP6
PRP43
Gene
Z_Shift_K_Doxo_HLD
Z_Im_K_Doxo_HLD
Z_Shift_K_Doxo_EG
Z_Im_K_Doxo_EG
Z_Shift_L_Doxo_HLD
Z_Im_L_Doxo_HLD
Z_Shift_L_Doxo_EG
Z_Im_L_Doxo_EG

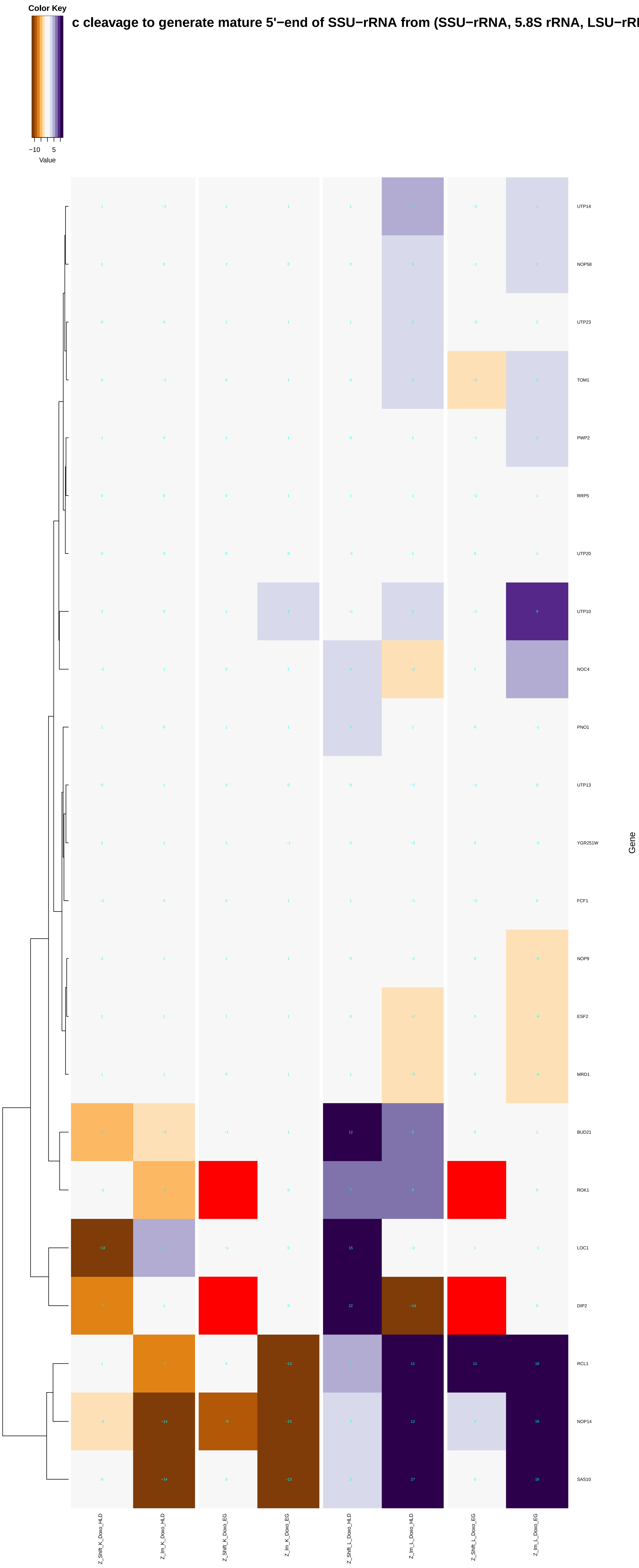
c cleavage to generate mature 5'−end of SSU−rRNA from (SSU−rRNA, 5.8S rRNA, LSU−rRI
Color Key
−10
5
Value
UTP14
NOP58
UTP23
TOM1
PWP2
RRP5
UTP20
UTP10
NOC4
PNO1
UTP13
YGR251W
FCF1
NOP9
ESF2
MRD1
BUD21
ROK1
LOC1
DIP2
RCL1
NOP14
SAS10
Gene
Z_Shift_K_Doxo_HLD
Z_Im_K_Doxo_HLD
Z_Shift_K_Doxo_EG
Z_Im_K_Doxo_EG
Z_Shift_L_Doxo_HLD
Z_Im_L_Doxo_HLD
Z_Shift_L_Doxo_EG
Z_Im_L_Doxo_EG

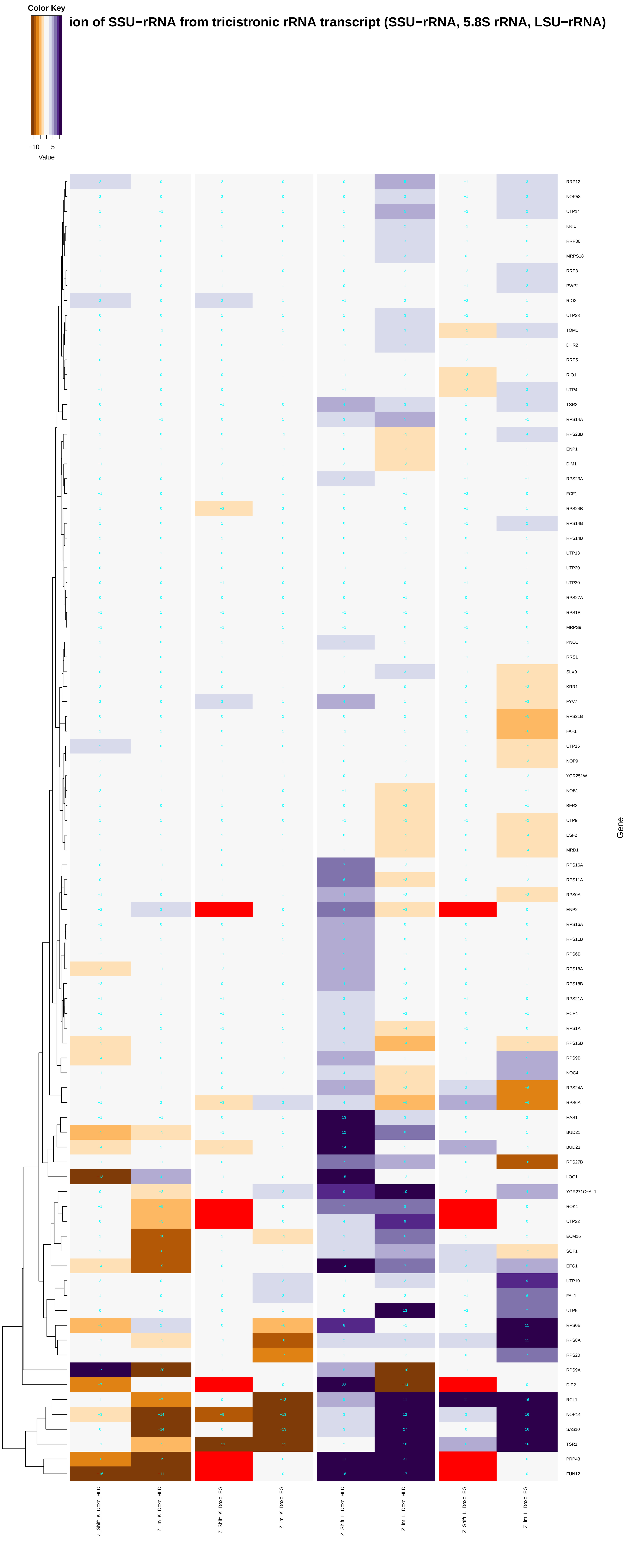
ion of SSU−rRNA from tricistronic rRNA transcript (SSU−rRNA, 5.8S rRNA, LSU−rRNA)
Color Key
−10
5
Value
RRP12
NOP58
UTP14
KRI1
RRP36
MRPS18
RRP3
PWP2
RIO2
UTP23
TOM1
DHR2
RRP5
RIO1
UTP4
TSR2
RPS14A
RPS23B
ENP1
DIM1
RPS23A
FCF1
RPS24B
RPS14B
RPS14B
UTP13
UTP20
UTP30
RPS27A
RPS1B
MRPS9
PNO1
RRS1
SLX9
KRR1
FYV7
RPS21B
FAF1
UTP15
NOP9
YGR251W
NOB1
BFR2
UTP9
ESF2
MRD1
RPS16A
RPS11A
RPS0A
ENP2
RPS16A
RPS11B
RPS6B
RPS18A
RPS18B
RPS21A
HCR1
RPS1A
RPS16B
RPS9B
NOC4
RPS24A
RPS6A
HAS1
BUD21
BUD23
RPS27B
LOC1
YGR271C−A_1
ROK1
UTP22
ECM16
SOF1
EFG1
UTP10
FAL1
UTP5
RPS0B
RPS8A
RPS20
RPS9A
DIP2
RCL1
NOP14
SAS10
TSR1
PRP43
FUN12
Z_Shift_K_Doxo_HLD
Z_Im_K_Doxo_HLD
Z_Shift_K_Doxo_EG
Z_Im_K_Doxo_EG
Z_Shift_L_Doxo_HLD
Z_Im_L_Doxo_HLD
Z_Shift_L_Doxo_EG
Z_Im_L_Doxo_EG
Gene

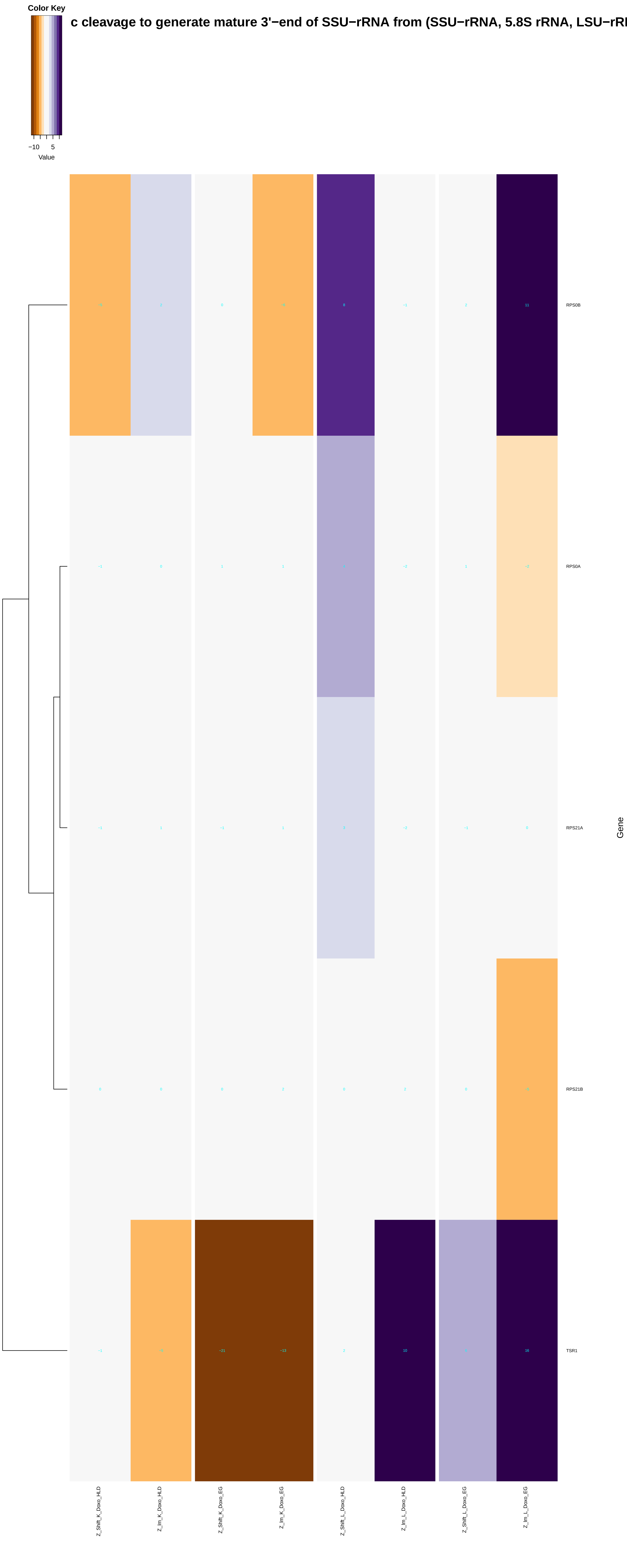

Color Key
−10
5
Value
c cleavage to generate mature 3'−end of SSU−rRNA from (SSU−rRNA, 5.8S rRNA, LSU−rR
RPS0B
RPS0A
RPS21A
RPS21B
TSR1
Gene
Z_Shift_K_Doxo_HLD
Z_lm_K_Doxo_HLD
Z_Shift_K_Doxo_EG
Z_lm_K_Doxo_EG
Z_Shift_L_Doxo_HLD
Z_lm_L_Doxo_HLD
Z_Shift_L_Doxo_EG
Z_lm_L_Doxo_EG

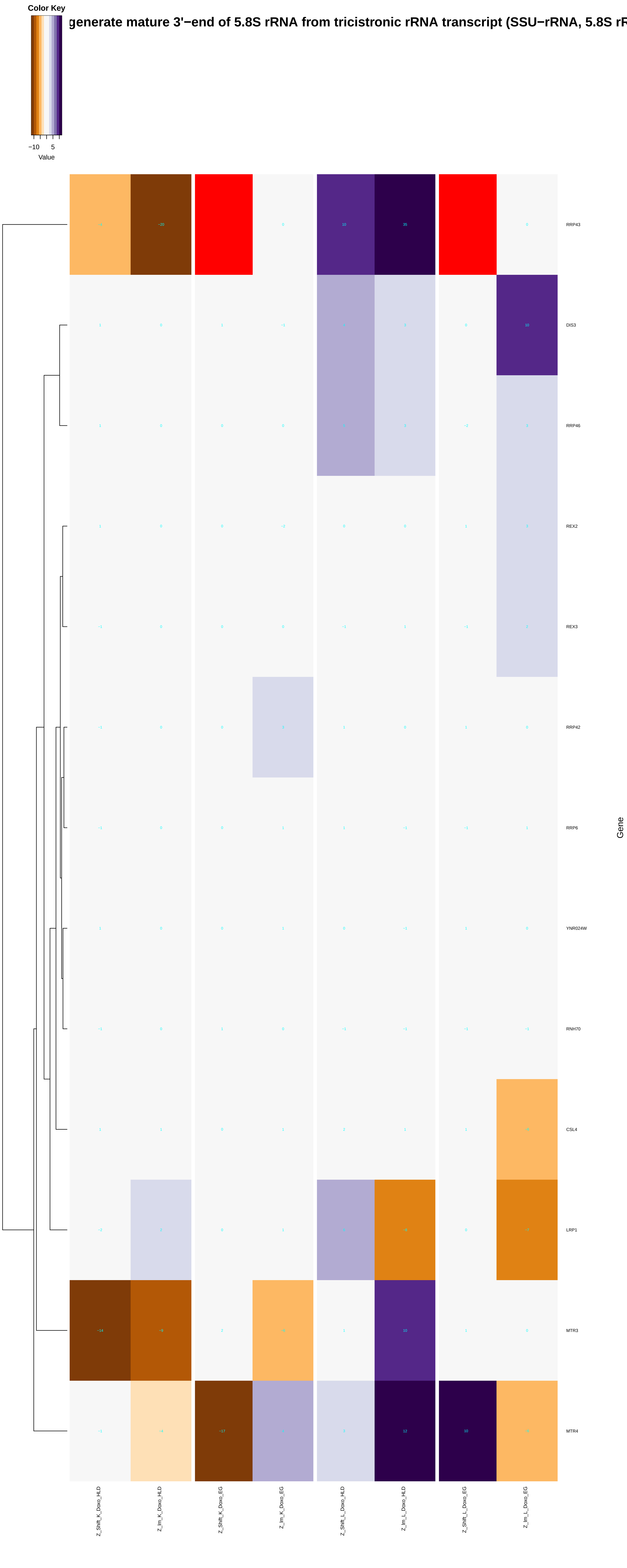

Color Key
−10
5
Value
generate mature 3'−end of 5.8S rRNA from tricistronic rRNA transcript (SSU−rRNA, 5.8S rR
RRP43
DIS3
RRP46
REX2
REX3
RRP42
RRP6
YNR024W
RNH70
CSL4
LRP1
MTR3
MTR4
Gene
Z_Shift_K_Doxo_HLD
Z_lm_K_Doxo_HLD
Z_Shift_K_Doxo_EG
Z_lm_K_Doxo_EG
Z_Shift_L_Doxo_HLD
Z_lm_L_Doxo_HLD
Z_Shift_L_Doxo_EG
Z_lm_L_Doxo_EG

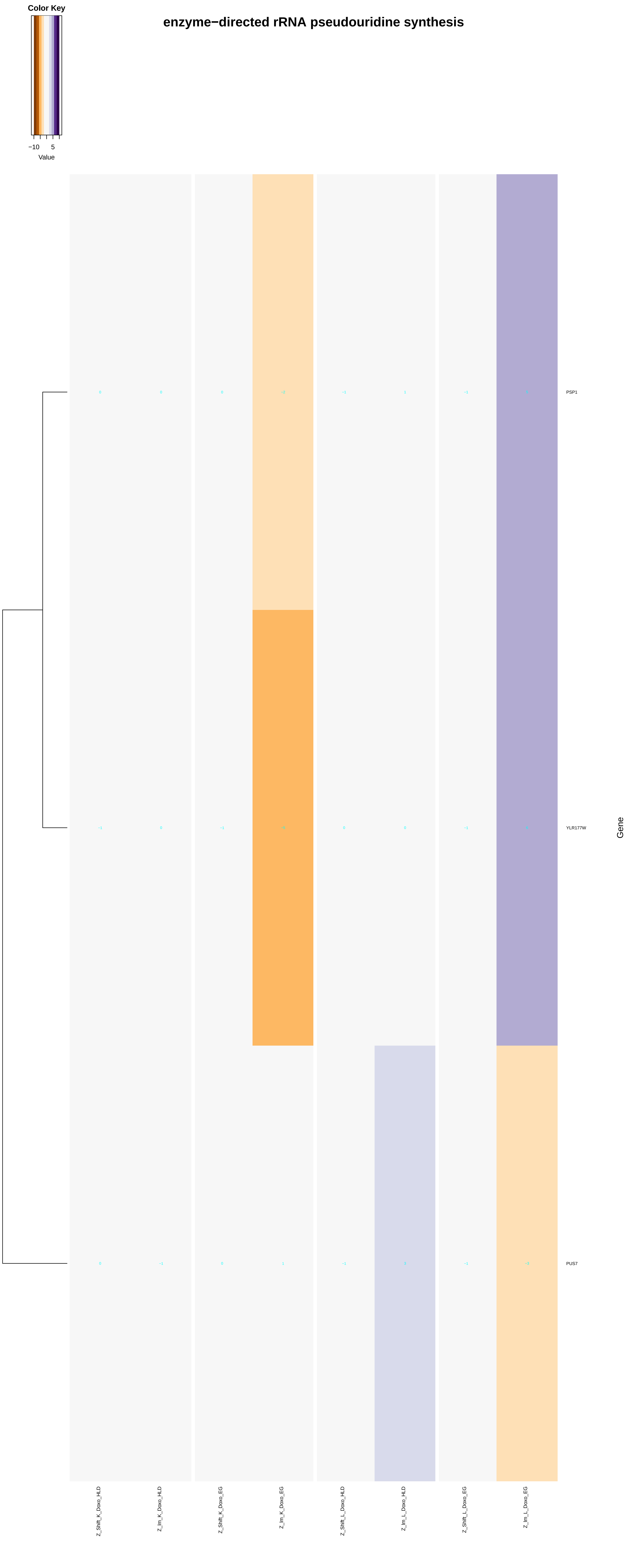

enzyme−directed rRNA pseudouridine synthesis
Color Key
−10
5
Value
PSP1
YLR177W
PUS7
Gene
Z_Shift_K_Doxo_HLD
Z_Im_K_Doxo_HLD
Z_Shift_K_Doxo_EG
Z_Im_K_Doxo_EG
Z_Shift_L_Doxo_HLD
Z_Im_L_Doxo_HLD
Z_Shift_L_Doxo_EG
Z_Im_L_Doxo_EG

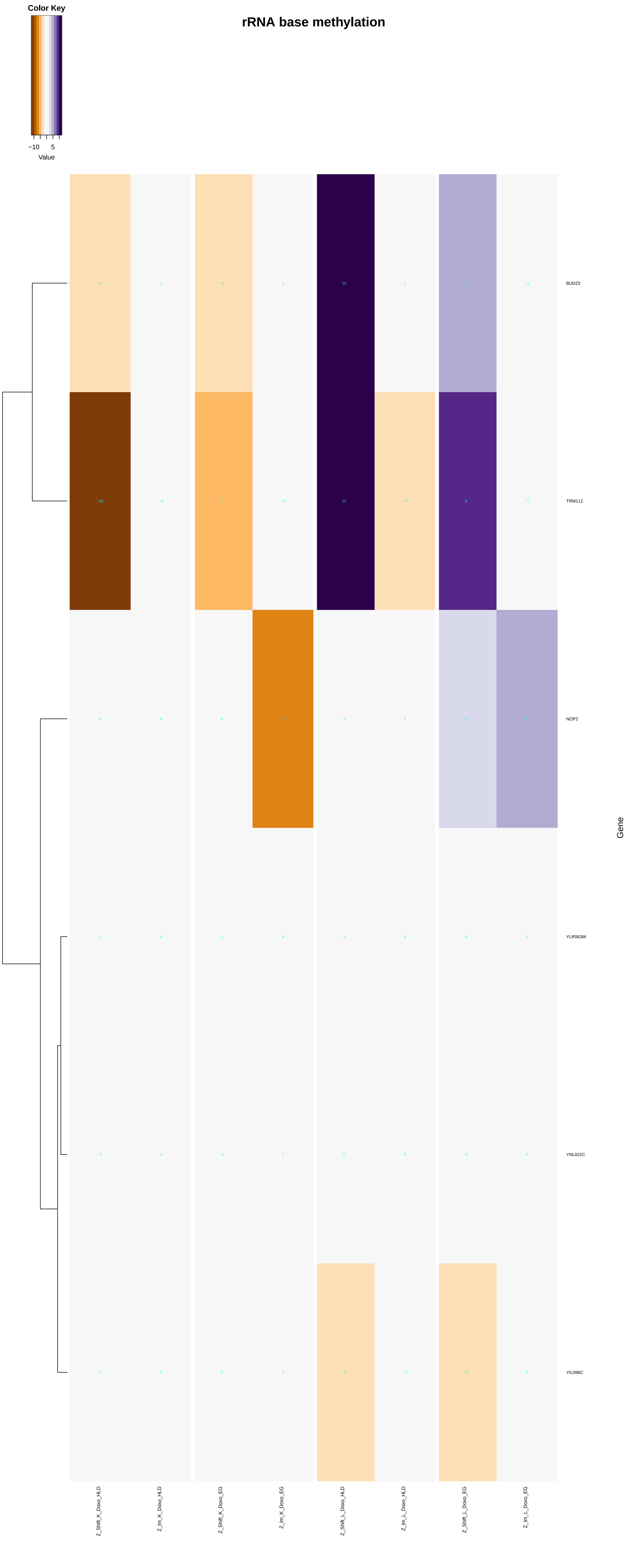

rRNA base methylation
Color Key
−10
5
Value
BUD23
TRM112
NOP2
YLR063W
YNL022C
YIL096C
Gene
Z_Shift_K_Doxo_HLD
Z_Im_K_Doxo_HLD
Z_Shift_K_Doxo_EG
Z_Im_K_Doxo_EG
Z_Shift_L_Doxo_HLD
Z_Im_L_Doxo_HLD
Z_Shift_L_Doxo_EG
Z_Im_L_Doxo_EG
